# High functional diversity in deep‐sea fish communities and increasing intraspecific trait variation with increasing latitude

**DOI:** 10.1002/ece3.7871

**Published:** 2021-07-08

**Authors:** Elisabeth M. V. Myers, Marti J. Anderson, Libby Liggins, Euan S. Harvey, Clive D. Roberts, David Eme

**Affiliations:** ^1^ New Zealand Institute for Advanced Study (NZIAS) Massey University Auckland New Zealand; ^2^ School of Natural and Computational Sciences Massey University Auckland New Zealand; ^3^ Auckland War Memorial Museum Tāmaki Paenga Hira Auckland New Zealand; ^4^ School of Molecular and Life Sciences Curtin University Bentley WA Australia; ^5^ Museum of New Zealand Te Papa Tongarewa Wellington New Zealand; ^6^ IFREMER Unité Ecologie et Modèles pour l’Halieutique Nantes France

**Keywords:** biodiversity, biotic interactions, deep‐sea fishes, depth gradient, environmental filtering, functional trait, morphology, niche partitioning

## Abstract

Variation in both inter‐ and intraspecific traits affects community dynamics, yet we know little regarding the relative importance of external environmental filters versus internal biotic interactions that shape the functional space of communities along broad‐scale environmental gradients, such as latitude, elevation, or depth. We examined changes in several key aspects of functional alpha diversity for marine fishes along depth and latitude gradients by quantifying intra‐ and interspecific richness, dispersion, and regularity in functional trait space. We derived eight functional traits related to food acquisition and locomotion and calculated seven complementary indices of functional diversity for 144 species of marine ray‐finned fishes along large‐scale depth (50–1200 m) and latitudinal gradients (29°–51° S) in New Zealand waters. Traits were derived from morphological measurements taken directly from footage obtained using Baited Remote Underwater Stereo‐Video systems and museum specimens. We partitioned functional variation into intra‐ and interspecific components for the first time using a PERMANOVA approach. We also implemented two tree‐based diversity metrics in a functional distance‐based context for the first time: namely, the variance in pairwise functional distance and the variance in nearest neighbor distance. Functional alpha diversity increased with increasing depth and decreased with increasing latitude. More specifically, the dispersion and mean nearest neighbor distances among species in trait space and intraspecific trait variability all increased with depth, whereas functional hypervolume (richness) was stable across depth. In contrast, functional hypervolume, dispersion, and regularity indices all decreased with increasing latitude; however, intraspecific trait variation increased with latitude, suggesting that intraspecific trait variability becomes increasingly important at higher latitudes. These results suggest that competition within and among species are key processes shaping functional multidimensional space for fishes in the deep sea. Increasing morphological dissimilarity with increasing depth may facilitate niche partitioning to promote coexistence, whereas abiotic filtering may be the dominant process structuring communities with increasing latitude.

## Graphical abstract

We quantified in situ inter‐specific and intra‐specific variation for deep‐sea fishes, providing novel methodology to partition community‐level trait variation into within and among species components in a multi‐trait context. Increasing morphological dissimilarity with depth may facilitate niche partitioning, promoting coexistence, whereas abiotic filtering may be the dominant process structuring communities across latitude.

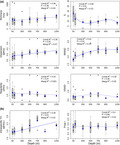

## 1 INTRODUCTION

Studying biodiversity across large‐scale environmental gradients plays a key role in aiding scientists to understand potential mechanisms shaping species’ distributions. Analyses of taxonomic diversity (*e.g*., species richness) are useful, but a more integrative understanding and insights regarding potential mechanisms driving biodiversity and ecosystem services can be obtained through analyses of phylogenetic diversity and functional diversity (Díaz et al., 2016; Swenson, 2011). Previous studies have documented a general decrease in species richness with increasing latitude, elevation, and depth (Costello & Chaudhary, 2017; Gaston, 2000; Hillebrand, 2004). However, functional diversity displays a variety of different patterns along gradients (Lamanna et al., 2014; Mouillot, Bellwood, et al., 2013; Stuart‐Smith et al., 2013; Villéger et al., 2010). Furthermore, trends in functional diversity will depend on the particular traits that are measured and the indices that are calculated from these.

Functional diversity is inherently multivariate, where each trait is a variable, and species (or individuals) occupy a particular position in multivariate trait space. There are many ways to measure functional diversity (Laliberté & Legendre, 2010; Mouchet et al., 2010; Villéger et al., 2008), and each metric may capture a different aspect of functional diversity. For example, one might measure the functional hypervolume covered by a set of species (“space”) or the packing (*i.e*., the proximity of neighboring species) within this volume (Lamanna et al., 2014; Swenson & Weiser, 2014). Patterns in these different aspects of functional diversity along environmental gradients vary, depending on the focal taxa and traits included, as well as the resolution and spatial scale of the study. For example, both functional richness and species richness were found to decline with increasing latitude for plants in the New World (Lamanna et al., 2014). In contrast, high species richness for birds at low elevations was characterized by a greater density (packing) of species in functional space rather than an increase in the volume (functional richness) of the space occupied (Pigot et al., 2016). Simultaneous analysis of multiple functional diversity measures, along with species richness, yields more comprehensive biodiversity information and insight into the potential processes driving community assembly.

External processes, such as abiotic filtering, and internal processes operating within the community, such as density‐dependent interactions (*e.g*., competition), work in tandem to shape functional diversity (Kraft, Adler, et al., 2015; Kraft, Godoy, et al., 2015; Swenson & Weiser, 2014; Violle et al., 2012). Abiotic filtering is assumed to select the number or type of functional strategies adapted to the environmental conditions encountered by a community (*i.e*., a species pool), determining its volume (or functional richness), whereas internal community processes such as competition are generally expected to affect the packing (or density) of species or individuals within the functional volume occupied by the community (Swenson & Weiser, 2014). For example, increasing competition for limited resources in environmentally harsh conditions is expected to decrease the packing of taxa in a functional space, whereby similar species are excluded from the community (Swenson & Weiser, 2014). Similarly, both external and internal drivers can also affect intraspecific functional variation (Violle et al., 2012).

Variation in traits among individuals within a species may be particularly important in species‐poor regions, for taxa with narrow geographic ranges, or that live in stressful environmental conditions (Hoffmann & Merilä, 1999; Siefert et al., 2015). For many species, the deep sea represents an example of both a species‐poor environment, and one with stressful environmental conditions (Priede, 2017), providing a unique opportunity to investigate patterns of inter‐ and intraspecific functional diversity along an increasing stress gradient. Disentangling individual‐level variability versus species‐level variation in functional trait space, including along environmental gradients, can provide novel insights into how communities are structured and maintained (Bolnick et al., 2011; Violle et al., 2012, 2014).

**FIGURE 1:**
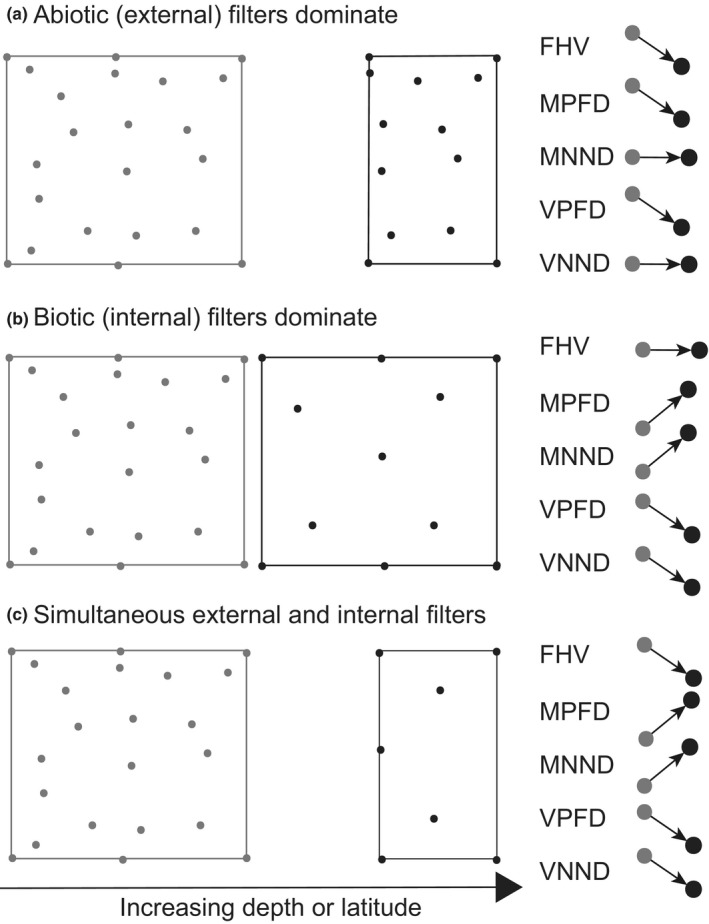
Schematic diagram of three conceptual models of changes in functional space (and functional diversity metrics) when moving from a relatively benign (gray), to a more extreme environment (black). Points represent species (or individuals), and the squares represent the bounds of functional space. (a) Abiotic filters cause a decrease in functional volume, but species packing remains unchanged. (b) Biotic filters, such as increased competition for limited resources, do not affect volume, but decrease the packing of species (or individuals) in functional space. (c) Harsh abiotic conditions, decreased resources, and increased competition combine to yield a decrease in volume and also in the packing of species. FHV, functional hypervolume; MPFD, mean pairwise functional distance; MNND, mean nearest neighbor distance; VPFD, variance in pairwise functional distance; VNND, variance in nearest neighbor distance

The depth gradient is one of the steepest environmental gradients on earth, yet is one of the least studied in terms of functional diversity. Changes with increasing oceanic depth are dramatic, including decreasing light, temperature, dissolved oxygen, food, and increasing pressure (Priede, 2017). These changes strongly influence the spatial distribution of species, their functions, and morphologies (Mindel et al., 2016; Myers et al., 2019; Zintzen et al., 2017). One of the most striking patterns is a decline in species richness with increasing depth. Assuming that a transition from shallow to deep waters or from subtropical to sub‐Antarctic latitudes represents a progression from a benign environment to an abiotically harsher, more extreme environment, we generated three contrasting conceptual models (a‐c) regarding expected functional changes in communities along these gradients. Each model yields specific predictions (hypotheses) regarding expected changes in functional diversity indices, indicative of the strength of influence of abiotic (external) versus biotic (internal) potential drivers (Figure 1).

(a) Abiotic filtering will be stronger in harsh environments, such as along increasing depth or latitude gradients (Swenson, 2011). This will lead to a decrease in functional hypervolume, but species packing should remain constant (Figure 1a). (b) Biotic interactions, such as competition for limited resources, will intensify with increasing depth or latitude. These interactions will decrease the packing of species (or individuals) in functional space, but the overall volume of the functional space will remain unchanged (Figure 1b). (c) Both abiotic filtering and biotic filtering will jointly affect functional diversity, decreasing both the volume and the packing of species (or individuals) in deeper or higher latitude regions (Figure 1c).

Here, we tested these predictions by quantifying patterns of change in functional diversity for 144 species of marine ray‐finned fishes along large‐scale depth (50–1,200 m) and latitudinal gradients (29°–51° S) in New Zealand waters. We obtained morphological measurements of individual fishes in situ using Baited Remote Underwater stereo‐Video systems (stereo‐BRUVs), allowing quantification of both interspecific trait variation and intraspecific trait variation. We focused on 8 functional traits related to food acquisition and locomotion and calculated 7 complementary functional diversity indices to examine their relationships with depth and latitude using univariate and multivariate approaches. By examining trends for a suite of different functional diversity metrics versus depth and latitude, we were able to characterize, more specifically, the *type* of change occurring in the functional space, and thus begin to differentiate among competing underlying models that might explain functional variation along these large‐scale gradients.

## 2 MATERIALS AND METHODS

### 2.1 Fish community data

We observed 144 species of marine ray‐finned fishes (Class Actinopterygii) on Baited Remote Underwater stereo‐Video (stereo‐BRUV) footage. Stereo‐BRUVs were deployed in a stratified random sampling design, with *n* = 5–7 replicate units at each of seven targeted depths: 50, 100, 300, 500, 700, 900, and 1200 m, within each of seven locations spanning 21° of latitude in New Zealand waters. In total, the footage from 329 deployments (3 hr each) across 47 depth‐by‐location cells was analyzed. For further details regarding the sampling design and stereo‐BRUV methodology, see Zintzen et al. (2012) and Zintzen et al. (2017).

### 2.2 Measurements from fish and derivation of traits

Stereo‐BRUVs are typically used to estimate relative abundance and biomass of fishes (Cappo et al., 2003), but can also be used to make fine‐scale point‐to‐point measurements of distances between specified morphological features (*e.g*., Harvey et al., 2003). To ensure the accuracy and precision of measurements, stereo‐BRUVs were calibrated before and after deployments (Boutros et al., 2015). Measurements were made using EventMeasure software (www.seagis.com.au) with the following rules: Individuals must be within 7 m of stereo‐BRUVs, have a maximum root‐mean‐square error of 20 mm (a quality measure indicating accuracy), and have a maximum precision‐to‐length ratio of 10% (Harvey & Shortis, 1998).

We identified 15 morphological measurements on the basic body plan for fishes to calculate functional traits pertaining to locomotion and food acquisition (Supplement 1, Figure S1; and Villéger et al., 2010). For each stereo‐BRUV deployment, we aimed to measure at least one full “set” of 15 measurements for at least one individual of each species present. Care was taken to prevent measuring the same individual twice. To get the most accurate measurements, individuals were preferentially chosen according to: (a) proximity to the camera, (b) unobscured view, (c) body position being side‐on/perpendicular to the camera, and (d) unconstrained configuration of morphological features.

From the raw morphological measurements, we derived eight functional traits (Table 1). Locomotion was captured by traits documenting eye position, pectoral fin position, caudal peduncle throttling, and elongation, while food acquisition traits included eye size, oral gape position, and jaw length/head length. Body size (considered to be a universal trait, Bellwood et al. (2019)) was measured as total length.

**TABLE 1:** Definitions of eight functional traits derived from raw morphological length measurements of individual fishes (see Figure S1 for an illustration)

	Functional Trait	Calculation	Ecological relevance
Universal trait	Total body length	TL	Proxy for overall body size; indication of trophic level or relative food web position
Food acquisition	Eye size*	Ed/Hd	Prey detection
	Oral gape position*	Mo/Hd	Feeding method in the water column
	Jaw length	$\frac{1}{2}$(Uj +Lj)/Hl	Proxy for size of oral gape; indication of the size of potential prey
Locomotion	Elongation	TL/mBd	Indication of overall body shape; greater elongation indicates steady swimming ability (Claverie & Wainwright, 2014)
	Eye position*	Eh/Hd	Indication of vertical position in the water column
	Caudal peduncle throttling*	CFd/CPd	Indication of the efficiency of caudal propulsion; reduction of drag
	Pectoral fin position*	PFi/PFb	Indication of maneuverability using pectoral fins

Traits adapted from Villéger et al. (2010) are identified by an asterisk.

Abbreviations: CFd, caudal fin depth; CPd, caudal peduncle depth; Ed, maximum eye diameter; Eh, eye height; Hd, head depth; Hl, head length; Lj, lower jaw length; mBd, maximum body depth; Mo, mouth open; PFb, body depth at pectoral fin base; PFi, pectoral fin insertion; TL, total body length; Uj, upper jaw length.

### 2.3 Data imputation

Complete morphological measurement data were not available for all of the species observed on the stereo‐BRUV footage. To fill in missing trait information, we followed a two‐step procedure. First, to guarantee an estimate of every trait for every species seen on the stereo‐BRUV footage, we obtained raw morphological measurements directly from *n* = 291 well‐preserved museum specimens (belonging to 144 species) from the National Fish Collection at the Museum of New Zealand Te Papa Tongarewa, Wellington (see Myers et al. (2019) and Myers et al. (in press) for details, including species list and voucher registrations). Next, for individuals missing 3 or fewer morphological measurements (out of 15), and for which there were 5 or more occurrences of that species with complete trait information across the dataset (*n* = 877; 586 rows of data from the stereo‐BRUV footage, and 291 from the museum dataset), we imputed missing values. This was done using a random‐forest machine‐learning model built from all conspecific individuals across the full study design that had the full set of 15 morphological measurements. The random‐forest approach handles complex nonlinear relationships, is computationally fast, and estimates imputation error (Penone et al., 2014; Stekhoven & Bühlmann, 2011). We used the “missForest” R package (Stekhoven & Bühlmann, 2011) and set the *maxiter* argument to 20 iterations and the *ntree* to 300 decision trees. We performed 20 runs and used the averaged output to obtain a single imputed value. This data imputation increased our stereo‐BRUV trait dataset by 136 to yield a total of 722 individuals that had a complete set of 15 morphological measurements for subsequent analysis.

### 2.4 Choice of individuals to use to represent individual species’ traits using a hierarchical approach

We analyzed data on the basis of the species present (observed in video footage) within each of the 47 depth‐by‐location cells. There were 144 species recorded and 509 species‐by‐cell occurrences (many species naturally occurred in more than one cell). Our original dataset was comprised of a complete set of 15 raw morphological measurements for a total of 722 individuals (136 of these required some random‐forest imputation, and missing traits were remeasured for four individuals) obtained directly from Stereo‐BRUV footage. From this original dataset, two datasets were generated: one for all species‐level functional metrics (*i.e*., FHV, MPFD, MNND, VPFD, and VNND; see Section 2.5 below) and one for all metrics focusing on intraspecific trait variability (*i.e*., MPFD.I and Prop.I; see Section 2.6 below). To generate the first dataset, we created 100 tables of 509 unique species‐by‐cell occurrences (rows) for the 8 traits (columns) by randomly drawing 1 individual from the list of all complete individuals for each species. To maintain any spatial structures in trait variability as well as possible, we drew an individual for each species within each cell from conspecific individuals that were (in order of preference): (a) within that depth‐by‐location cell, (b) at the same depth, or (c) from anywhere within the Stereo‐BRUV study design (*n* = 722) or (d) from a museum specimen (*n* = 291). All species‐level functional metrics were calculated for each replicate table, and we calculated the mean across all 100 tables for every metric for subsequent analyses.

For metrics focusing on intraspecific trait variability, we used data solely from the in situ stereo‐BRUV footage (*i.e*., the dataset comprising 722 individuals). Due to the inability to measure every species observed on the stereo‐BRUVs, this dataset represents a reduced number of species (62 out of 144) and cells (43 out of 47). Within this dataset, we were able to measure intraspecific trait variability for 42 species (2 or more individuals per species per cell). There were, on average, 3.34 species per cell (min = 1, max = 10, sd = 1.86) and 4.32 individuals per species per cell (min = 2, max = 15, sd = 2.5) to measure the intraspecific trait variability.

### 2.5 Functional metrics

All functional metrics were calculated using 8 normalized continuous traits. We calculated the following species‐level metrics for each depth‐by‐latitude cell, for each of the 100 species‐by‐trait (509 x 8) data matrices after calculating Euclidean distances: (a) mean pairwise functional distance (MPFD; (Clarke & Warwick, 1998; Somerfield et al., 2008; Swenson, 2014), (b) mean nearest neighbor distance (MNND; Swenson & Weiser, 2014), (c) variance in pairwise functional distance (VPFD; adapted from Clarke and Warwick (2001) and Somerfield et al. (2008)), and (d) variance in nearest neighbor distance (VNND; Swenson (2014).

MPFD is the functional analogue to average taxonomic distinctness (Clarke & Warwick, 1998) and mean phylogenetic pairwise distance (Swenson, 2014). It is highly correlated with functional dispersion (Laliberté & Legendre, 2010) and Rao's quadratic entropy (Botta‐Dukát, 2005), but is independent of species richness and only weakly influenced by outliers. MNND has been used previously in both phylogenetic (Webb et al., 2002) and functional contexts (Pigot et al., 2016; Swenson & Weiser, 2014). It measures the average minimum distance in the functional strategies of co‐occurring species and has previously been used to estimate functional originality (Leitao et al., 2016; Mouillot, Graham et al., 2013).

VPFD quantifies the regularity of the distances among species in the functional space. This measure was originally proposed by Clarke and Warwick (2001) in a taxonomic setting. Here, we calculate it in functional space (as suggested by Somerfield et al., 2008), and it is independent of species richness and MPFD. Similarly, VNND also quantifies regularity, but focuses on functional similarity between nearest neighbors. It was proposed by Swenson (2014) in a phylogenetic context, but we calculated it here in functional space.

We also performed principal component analysis (PCA) on the normalized traits in order to calculate functional hypervolume (FHV; Blonder et al., 2014, 2018). FHV was calculated using the first 4 principal component axes (which accounted for 70.2%–74.4% of the total variation in the 8D functional trait space across the 100 species‐by‐trait tables). We did not retain all 8 dimensions due to difficulties associated with calculating FHV when few species were present. FHV has been used as a proxy to estimate niche space, including high‐dimensional, irregular spaces (Cooke et al., 2019; Lamanna et al., 2014). The Gaussian kernel density estimation method was chosen to form a “loose wrap” around the data (Blonder et al., 2018). We used a quantile threshold of 0.05, retaining 95% of the total probability density, and estimated the bandwidth vector using the Silverman method (Blonder et al., 2018).

### 2.6 Quantifying intraspecific trait variability

Next, we calculated individual‐level trait variation and determined its contribution toward total functional trait variation (among species and among individuals), within each cell. From the original base dataset (722 individuals), we first calculated mean pairwise functional distance (MPFD.I) directly, considering only the intraspecific distances. In addition, partitioning was done by performing a PERMANOVA on the Euclidean distances among all complete individuals separately within each cell. Different species were treated as different levels of the factor “Species,” and individuals within each species were treated as replicates in a one‐factor design. The expectations of mean squares were used to calculate functional multivariate analogues to the classical unbiased ANOVA estimators’ univariate variance components. More specifically,

$$\sigma_{I}^{2}={\text{MS}}_{\text{Res}}\;\text{and}\;\sigma_{S}^{2}=\frac{\left({\text{MS}}_{\text{Species}}‐{\text{MS}}_{\text{Res}}\right)}{n_{0}}$$

where $\sigma_{I}^{2}$ is the estimated individual‐level trait variance, $\sigma_{S}^{2}$ is the estimated species‐level trait variance, MS_Res_ is the residual mean square, MS_Species_ is the “Species” factor's mean square from the PERMANOVA partitioning, and $n_{0}$ is a divisor that allows for unequal numbers of individuals ($n_{i}$ per species *I* = 1,…*S*) within each cell, calculated as follows (Sokal and Rohlf (1981), p. 214):

$$n_{0}=\frac{1}{\left(a‐1\right)}\left\{\sum_{}^{a}n_{i}‐\left(\frac{\sum_{}^{a}n_{i}^{2}}{\sum_{}^{a}n_{i}}\right)\right\}$$

The proportion of total trait variation (within each cell) attributable to individual‐level variation (*i.e*., Prop.I) is then calculated as.

$$\text{Prop}\text{.I}=\frac{\sigma_{I}^{2}}{\sigma_{S}^{2}+\sigma_{I}^{2}}$$

This is the first time, to our knowledge, that individual‐level functional variation and species‐level functional variation have been partitioned and calculated in this way for full‐dimensional multivariate trait space. Importantly, the estimators $\sigma_{I}^{2}$ and $\sigma_{S}^{2}$ are independent of species richness. Also, while small or unbalanced sample sizes ($n_{i}$, the number of individuals per species) will necessarily affect the precision of the estimates (*i.e*., small numbers of individuals or species will yield more variable estimates), they will not, however, affect the accuracy of these measures, which are unbiased. There was very high correlation in the values of $\sigma_{I}^{2}$ and MPFD.I (*r* = 0.93), and in the values of $\sigma_{S}^{2}$ and MPFD (*r* = 0.89) (Figure S3), so we present results only for MPFD, MPFD.I, and Prop.I in what follows. Finally, note that Prop.I and MPFD.I could only be calculated when there were two or more individuals representing the same species within a depth‐by‐location cell.

Some previous approaches to disentangle intra‐ from interspecific trait variation for community‐level data (*e.g*., Lepš et al., 2011) have focused simply on differences between two weighted averages: (a) an average based on fixed species‐specific trait values and (b) an average based on trait values for each species that may vary across sites. However, consider a scenario where the mean quantities (a) and (b) above are quite similar to one another in value, hence produce approximately zero mean difference, yet variation in individual trait values is nevertheless quite high. This approach does not attempt to measure the actual variance in trait values among individuals, per se, nor does it produce statistically independent values for the intra‐ and interspecific components, whereas the PERMANOVA partitioning and calculation of variance components outlined above do achieve this.

The method described by de Bello et al. (2011) is aimed at quantifying functional turnover among habitats primarily for singular traits, rather than quantifying functional diversity within habitats and across multiple traits, which is our primary focus here. These authors do usefully mention the PERMANOVA approach and note its equivalences, where appropriate, with partitioning according to quadratic entropy diversity measures. They do not articulate or distinguish, however, the crucial additional calculations required to transform the raw additive sums of squares (arising directly from either a PERMANOVA or ANOVA partitioning) into estimates of independent variance components. The relative sizes of sums of squares from a partitioning will depend on their degrees of freedom, whereas variance components are unbiased estimators of variance derived from expectations of mean squares. Such components, calculated here in a functional context, can be compared across different factors (*e.g*., in hierarchical/nested designs; see Anderson et al., 2005) and are independent of sample size (numbers of species or individuals).

### 2.7 Statistical analysis

#### 2.7.1 Univariate models

For modeling, we considered depth and latitude as continuous predictor variables and included quadratic and cubic terms to allow for nonlinearities in response variables along these gradients. We chose to use a parametric model rather than a generalized additive model (GAM), because there were only 7 depth strata and 7 latitudinal bands. We consider that GAMs are better suited for data where there are more continuous x‐values along the gradient (independent variable) of interest. We normalized depth and latitude to ensure polynomial terms remained orthogonal. We analyzed all metrics described above as response variables; VPFD and VNND were transformed to $y^{\prime }=\log_{10}\left(\right)$ prior to analysis to improve normality. In order to explore the strength and generality of the potential relationship between the functional diversity metrics and either depth or latitude, we used linear mixed‐effects models, first treating depth as a fixed factor and latitude as a random factor, then treating latitude as fixed and depth as random (see Supplement 2, Tables S1–S2 for further details). The mixed model allows a test and quantification of the effects (if any) of a chosen fixed gradient of interest, over and above any potential variation in those effects (*i.e*., possible interaction) across a second (random) factor (*e.g*., as in Quintero & Jetz, 2018). In addition, we have considered depth and latitude as fixed factors and tested their interaction. We used a linear model to test this interaction when only the linear term for both depth and latitude was retained by the selection of the best linear mixed model. If at least one polynomial term was retained for depth or latitude, we used a GAM to assess the significance of a tensor product smooth term to test the nonlinearities in the interaction between depth and latitude. Despite GAM being better suited for a more continuous independent variable, when testing the nonlinearities in the interaction between depth and latitude, we have now included 47 depth‐by‐latitude combinations which is more in line with the use of GAM, rather than investigating the depth or latitude pattern alone considering only seven modalities. We constrained the smoothing terms for a maximum of 3 degrees of freedom to avoid overfitting. GAMs were performed with the mgcv R package (Wood, 2004).

#### 2.7.2 Multivariate analyses

To visualize changes in multiple functional metrics along depth and latitude gradients simultaneously, we did a metric multidimensional scaling (mMDS) ordination on (a) depth centroids and (b) latitude centroids (see Figure S4 for an mMDS ordination of all 47 depth‐by‐location cells). We superimposed bubbles corresponding to species richness and vectors to show partial correlations of functional metrics with mMDS axes. Three‐dimensional shade plots were obtained to visualize potential interactions between depth and latitude for each of FHV, MPFD, MNND, MPFD.I, and Prop.I (Figure S2). All multivariate analyses were done on the basis of Euclidean distances for *p* = 7 functional metric variables using PRIMER v7 (Clarke & Gorley, 2015) with the PERMANOVA+add‐on (Anderson et al., 2008).

## 3 RESULTS

**FIGURE 2:**
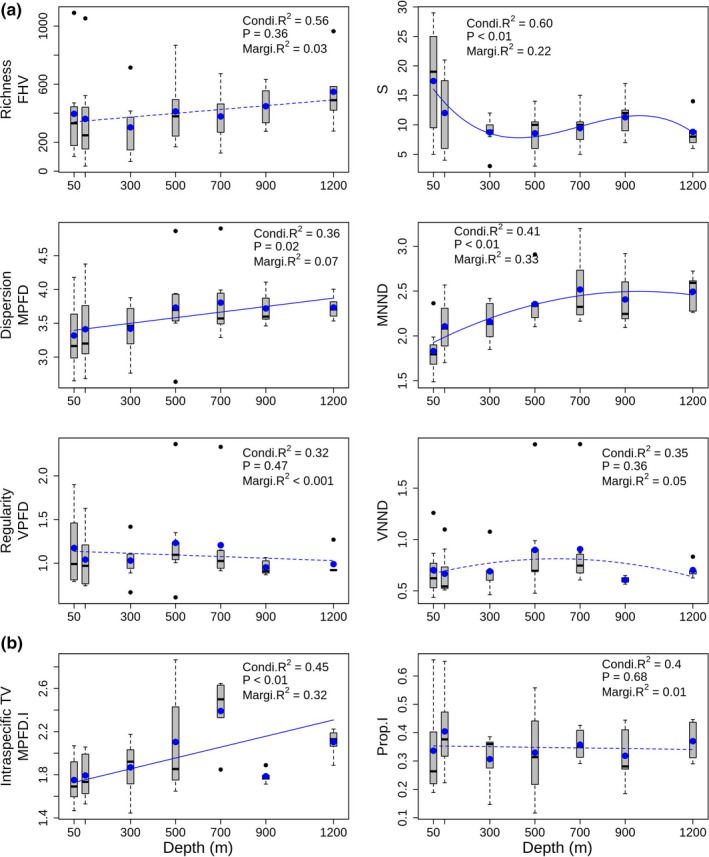
Relationships between functional diversity metrics (mean ± 1SE across 7 locations) and depth: (a) species‐level metrics (S = species richness) and (b) intraspecific trait variability metrics. Lines correspond to fitted values for the best model when depth was considered a fixed factor and latitude a random factor (see Table S1 in Supplementary Material). Solid lines show statistically significant trends (*p* < .05); dashed lines show trends that did not reach statistical significance at the 0.05‐level. The conditional *R* ^2^ (Condi.*R* ^2^) and marginal *R* ^2^ (Margi.*R* ^2^) are overlaid for each metric and represent variation explained by both the fixed and random effects jointly and variation explained only by the fixed effects, respectively (Nakagawa et al., 2017). Blue dots represent the average value per depth. Black horizontal bars, black dots, and boxes show the median, outliers, and interquartile range, respectively. Whiskers represent 1.5 times the interquartile range

**FIGURE 3:**
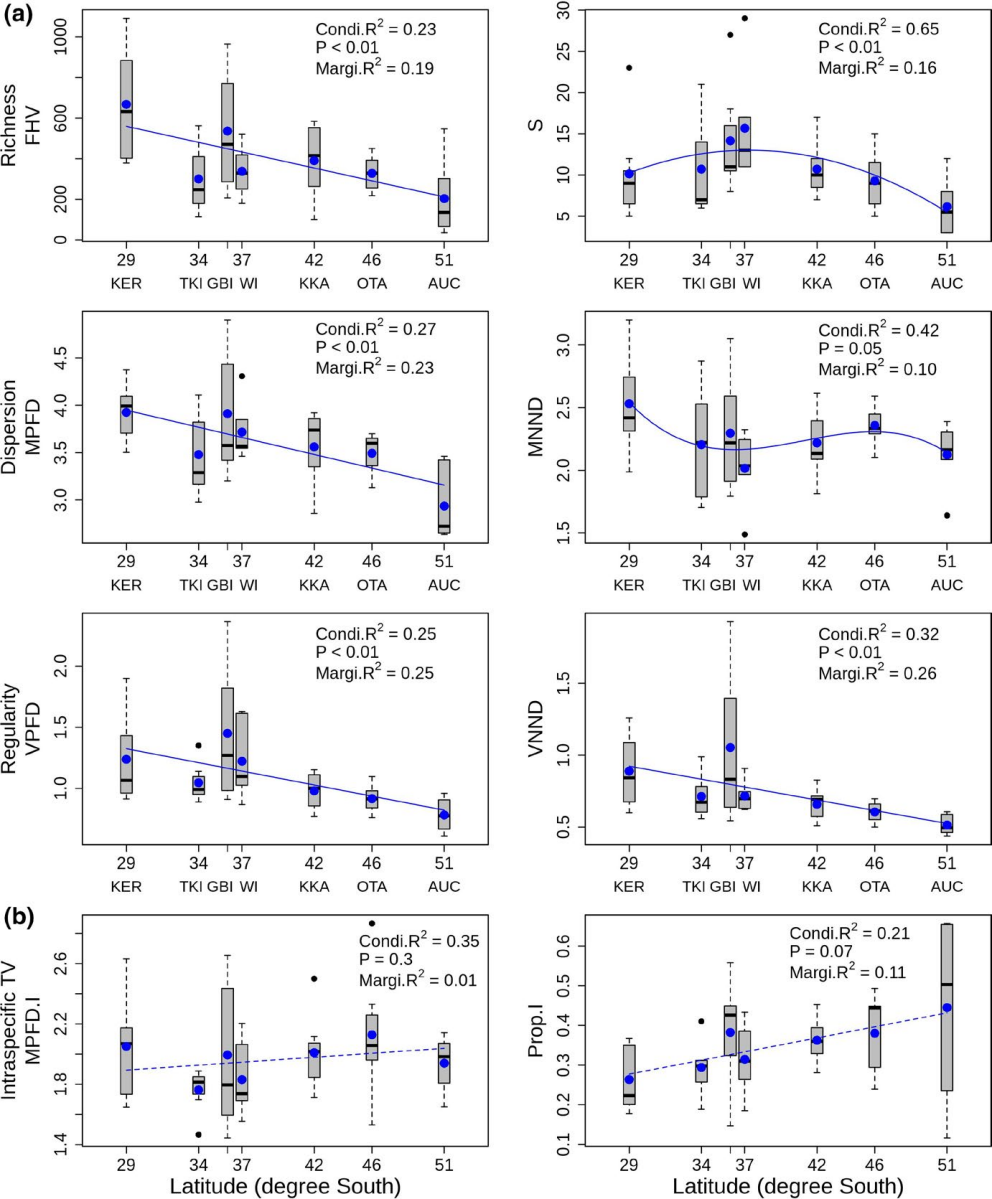
Relationships between functional diversity metrics (means ± 1SE across 7 depth strata) and latitude in degrees south (KER = Rangitāhua, Kermadec Islands, TKI = Three Kings Islands, GBI = Great Barrier Island, WI = Whakaari, White Island, KKA = Kaikōura, OTA = Otago, and AUC = Auckland Islands): (a) species‐level metrics; (S = species richness) and (b) intraspecific trait variability metrics. Lines show fitted values for the best model for each metric when latitude was considered a fixed factor, and depth a random factor (see Table S2 in Supplementary Material). Solid lines show statistically significant trends (*p* < .05); dashed lines show trends that did not reach statistical significance at the 0.05‐level. The conditional *R* ^2^ (Condi.*R* ^2^) and marginal *R* ^2^ (Margi.*R* ^2^) are overlaid for each metric and represent variation explained by both fixed and random effects jointly and variation explained only by the fixed effects, respectively (Nakagawa et al., 2017). Blue dots represent the average value per latitude. Black horizontal bars, black dots, and boxes show the median, outliers, and interquartile range, respectively. Whiskers represent 1.5 times the interquartile range

Functional hypervolume (FHV) was stable across the depth gradient, which was in line with our second prediction derived from our conceptual model (Figure 1b). The relationship between FHV and depth was not statistically significant (*p* =.36); however, FHV tended to generally increase with increasing depth, with the greatest volume found at the deepest depth (1200 m) (Figure 2a). Interestingly, the trends for functional richness (FHV) and species richness were decoupled, with species richness generally decreasing with increasing depth (Figure 2a). Functional dispersion metrics increased significantly with increasing depth, consistent with predictions arising from our second and third models (Figure 1b), with MPFD showing a clear break between shallow (50–300 m) and deep (500–1,200 m) areas (Table S1, *p* = .02), whereas MNND increased steeply from shallow to intermediate depths, followed by a plateau between 700 and 1,200 m (Table S1, *p* < .01 and *p <* .05 for the linear and quadratic terms, respectively). Regularity indices (VPFD and VNND; Figure 2a) neither increased nor decreased with increasing depth, but instead were characterized by high latitudinal variability at intermediate depths (500–700 m) and low variability at deeper depths (900–1,200 m). Intraspecific trait variability (MPFD.I) increased significantly with increasing depth (Table S1, *p* < .01), whereas Prop.I showed no clear trend with depth, explaining on average around 30%–40% of total trait variation across all depths (Table S1, Figure 2b). In general agreement with our first model (Figure 1a, abiotic filtering dominates), all interspecific metrics (except MNND) decreased monotonically with increasing latitude (Figure 3a). Regularity indices (VPFD and VNND) were highly variable at GBI compared to other locations. Although intraspecific trait variation (MPFD.I) did not increase significantly with latitude, the proportion of functional trait variation attributable to intraspecific differences (Prop.I) did increase significantly with increasing latitude, and was most variable at the southernmost latitude (AUC) (Table S2, Figure 3b). Latitudinal variation (as a random effect) exceeded variation attributable to the (fixed) effects of depth for FHV, MPFD, VPFD, VNND, and Prop.I, whereas depth effects exceeded latitudinal variation for MNND and MPFD.I (*cf*. Marginal *R* ^2^ and Conditional *R* ^2^ values, Table S1, see also Table S2). The interaction between depth and latitude was significant and positive for FHV, MPFD, VPFD, and negative for Prop.I (Table S3). Despite tensor products being nonsignificant for MNND and VNND a certain degree of interaction could be detected visually (see Figure S5 and Table S4).

**FIGURE 4:**
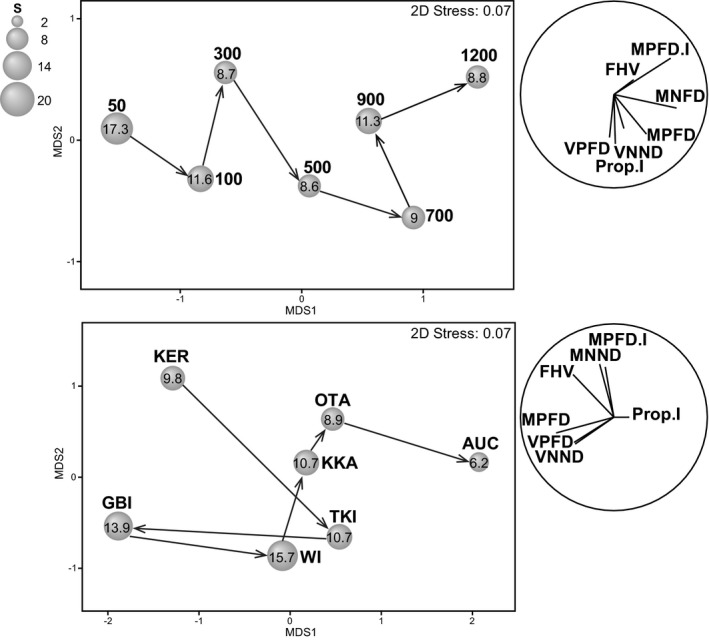
Metric multidimensional scaling (mMDS) ordination of normalized functional diversity metrics on the basis of Euclidean distances among (a) depth centroids (50 m to 1,200 m) and (b) location centroids. Overlaid arrows follow a shallow to deep, and north to south trajectory, respectively. Bubble sizes are proportional to mean species richness (also provided as a value inside each bubble). Vectors (right) show multiple partial correlations for each of *p =* 7 functional metrics with the mMDS axes

There were clear gradual changes in the functional diversity of fishes along the depth gradient from shallow to deep environments (*i.e*., from left to right along MDS axis 1 in Figure 4a). This was primarily characterized by increases in MNND and MPFD.I at deeper depths (Figure 4a). There were also clear sequential latitudinal changes in functional diversity metrics from north to south (*i.e*., from left to right along MDS axis 1 in Figure 4b). Northern locations were generally characterized by higher richness (FHV), dispersion (MPFD, MNND), and decreased regularity (*i.e*., higher values of VPFD and VNND), while southern locations tended to have higher intraspecific variation (MPFD.I, Prop.I). GBI, however, was clearly distinguishable from all other locations, having very high values of VPFD and VNND (hence, low regularity and a greater clumping of species across the functional space; Figure 4b, Figure S4).

## 4 DISCUSSION

This study examined several functional diversity metrics simultaneously, and also partitioned variation in functional space into intra‐ and interspecific components, enabling novel inferences regarding the relative contributions of external versus internal filters shaping fish communities along depth and latitude gradients. Functional alpha diversity increased with increasing depth and decreased with increasing latitude for New Zealand's ray‐finned marine fishes. More specifically, with increasing depth there were increases in (a) the dispersion and nearest neighbor distances among species within the trait space, and (b) intraspecific trait variation (MPFD.I). Interestingly, our results showed a decoupling of species and functional richness, whereby the overall functional hypervolume occupied by species remained stable along the depth gradient despite a decrease in species richness. Regularity indices were characterized by high latitudinal variability at intermediate depths (500–700 m) and low variability at deeper depths (900–1,200 m).

These results suggest that species occurring at deeper depths have diverged from one another to occupy distant morphological niches, with low levels of clustering, leading to a decrease in the *packing* of functional space with increasing depth. These results are consistent with our second conceptual model (Figure 1b) whereby biotic interactions, such as competition for limited resources, are the dominant processes shaping functional space at deeper depths. In addition, we found that functional hypervolume, functional dispersion, and functional regularity decreased with increasing latitude. These results are consistent with our first conceptual model (Figure 1a), in which environmental filtering is the dominant process. Interestingly, although species‐level metrics decreased with increasing latitude, individual‐level metrics increased (Figure 3), suggesting that there was a gradual change in the source of functional diversity, with intraspecific trait variability becoming increasingly important at higher latitudes.

The two gradients of depth and latitude showed contrasting patterns with respect to functional hypervolume (Figures 2, 3), which remained stable with increasing depth (the positive trend was not significant), but decreased with increasing latitude. The stability of the functional hypervolume metric across the depth gradient is a striking result because it provides a rare example of a mis‐match between species richness and functional richness; generally, when the number of species in a community increases, it is more likely to lead to a greater functional volume of that community (Villéger et al., 2008). The weak positive trend with depth, albeit nonsignificant, was an unexpected result and is certainly inconsistent with the idea that functions will be filtered more strongly in harsh environments (Swenson, 2011). Instead, abiotic conditions such as limited trophic resources and habitat availability, decreasing temperature, and increasing pressure may represent key selection pressures on individuals living in the deep sea (Ramirez‐Llodra et al., 2010). Species living in extreme conditions may be subject to greater disruptive selection and/or character displacement, potentially contributing to distinct morphologies, trait combinations, or functional strategies (Weiher & Keddy, 1995) that enable a greater variety of unique biotic adaptations for resource acquisition (Leitao et al., 2016). This may allow greater partitioning of limited resources, consistent with the limiting similarity hypothesis (MacArthur & Levins, 1967). Morphological dissimilarities among deep‐sea species reflect low niche overlap, which can promote coexistence in a low‐resource environment (Kumar et al., 2017). Decreasing functional hypervolume with increasing latitude, however, follows a more classical stress‐gradient hypothesis, whereby traits are filtered more strongly in harsh than in benign environments (Swenson, 2011; Weiher & Keddy, 1995). This pattern has been documented for plants versus latitude (Lamanna et al., 2014), for birds versus altitude (Pigot et al., 2016), and for macroinvertebrate assemblages versus depth (Ashford et al., 2018).

The packing, or density, of species within functional space decreased with increasing depth, as the mean distance between species and mean nearest neighbor distances both increased (Figure 2a). Previous work has shown that variance in pairwise phylogenetic distance (Eme et al., 2020) among fish species increases with depth in New Zealand waters. In contrast, we found that variance in functional distances among species was minimal at 900 and 1,200 m, suggesting species are evenly distributed in functional space (albeit comparatively widely), with nearest neighbors in functional space being far apart, despite the fact that phylogenetically, nearest neighbors are tightly clustered. Thus, it appears that species occurring at deeper depths diverge from one another functionally to occupy distant morphological niches. Morphological dissimilarities may help relax competition among species for limited resources (*i.e*., a stabilizing niche difference, sensu HilleRisLambers et al. (2012)) aiding in niche partitioning, and preventing the exclusion of inferior competitors (Swenson & Weiser, 2014). In trait‐based ecology, studies advocating measurement of intraspecific trait variability are becoming increasingly common (Des Roches et al., 2018; Siefert et al., 2015; Violle et al., 2012). This is the first time, to our knowledge, that multivariate variation across multiple functional traits has been partitioned into intra‐ and interspecific components using a hierarchical PERMANOVA approach (but see Albert et al., 2010; Jordani et al., 2019 for univariate examples using linear mixed models, and de Bello et al. (2011) for an example quantifying functional turnover among habitats primarily for singular traits using a PERMANOVA approach). We found an increase in intraspecific trait variability (MPFD.I; Figure 2b) with depth but, interestingly, the proportion of variance attributed to individual‐level variability was constant along the depth gradient. However, the proportion of total functional trait variation attributable to individual‐level variability increased with increasing latitude (Prop.I; Figure 3b), supporting the idea that intraspecific trait variation becomes important in species‐poor communities with narrow environmental breadth (Siefert et al., 2015). For example, the high‐latitude subantarctic Auckland Islands (AUC) had the highest value for Prop.I and was characterized by low species diversity, and a narrow temperature ranges from shallow to deeper environments (9.3–5.5°C). High levels of intraspecific phenotypic variability may contribute to the higher rates of speciation in fishes in the deep‐sea and at high latitudes (Rabosky et al., 2018), boosting the evolutionary potential of populations (Jump et al., 2009). Intraspecific variation begets speciation, which, in turn, begets interspecific variation (Darwin, 2009; Pfennig & Pfennig, 2010).

Despite intraspecific variation being at the core of evolutionary biology, community ecology has focused mostly on interspecific variations due to the lack of data on intraspecific variation for large communities. However, greater connections between evolutionary and ecological studies (Johnson & Stinchcombe, 2007; Mouquet et al., 2012), and the emergence of larger datasets aid the revival of studying intraspecific variation within community ecology (Siefert et al., 2015). Our intraspecific results were based on a limited number of individuals per species per site (4.32 on average) that should, ideally, be improved. However, measuring more individuals per species per site is not always possible depending on the species’ behavior (*e.g*., territorial or long‐ranging species). These findings, nevertheless, illustrate the importance of considering intraspecific variation in trait‐based studies at the community level.

Overall, our results, supporting inter‐ and intraspecific competition as a potential driver of niche partitioning, question the primary role of abiotic filtering in these harsh environments (Priede, 2017). Competition may have a greater role in shaping communities of the deep sea than previously thought. Our study has provided novel insights into how functional diversity changes along environmental gradients at the local (alpha) scale. We consider that future work should examine turnover and nestedness components of functional beta diversity to yield further potential insights into how ecological processes may structure communities. We also consider that functional traits from undersampled taxa *and* environments need additional study (Borgy et al., 2017). In addition, multivariate analyses of morphological traits should be extended to include behavioral traits, life‐history strategies, trophic positions, and/or physiological traits, for a more holistic measure of biologically relevant trait space (Bellwood et al., 2019; Violle et al., 2007).

## 5 CONCLUSIONS

These results suggest that interspecific and intraspecific competitions act as key processes shaping the functional diversity of fishes in the deep sea. Increasing morphological dissimilarity with increasing depth may help to facilitate niche partitioning and promote coexistence, whereas external abiotic filtering may be the dominant factor structuring communities with increasing latitude. In an era characterized by rapid and unprecedented change to deep‐sea environments, with increasing anthropogenic pressures from fishing, deep‐sea mining, and global climate change (Levin & Le Bris, 2015; Levin et al., 2016; Watson & Morato, 2013), understanding how functional diversity changes along large spatial gradients may help to predict potential responses of ecological communities to disturbances. In summary, this study quantified trait variation in marine fishes across broad‐scale depth and latitudinal gradients, shedding new light on the potential roles of abiotic filtering, biotic interactions, and niche partitioning, to further our understanding of the mechanisms underlying large‐scale patterns in biodiversity.

## CONFLICT OF INTEREST

The authors declare the absence of conflicts of interest.

## AUTHOR CONTRIBUTIONS

**Elisabeth M. V. Myers:** Conceptualization (equal); Data curation (lead); Formal analysis (equal); Investigation (equal); Methodology (equal); Project administration (lead); Visualization (lead); Writing‐original draft (lead); Writing‐review & editing (equal). **Marti J. Anderson:** Conceptualization (supporting); Formal analysis (supporting); Funding acquisition (lead); Investigation (supporting); Methodology (equal); Project administration (supporting); Resources (lead); Software (lead); Supervision (lead); Validation (supporting); Writing‐review & editing (supporting). **Libby Liggins:** Conceptualization (supporting); Funding acquisition (supporting); Investigation (supporting); Supervision (supporting); Validation (supporting); Writing‐review & editing (supporting). **Euan S. Harvey:** Resources (supporting); Supervision (supporting); Writing‐review & editing (supporting). **Clive D. Roberts:** Conceptualization (supporting); Funding acquisition (supporting); Resources (equal); Supervision (supporting); Writing‐review & editing (supporting). **David Eme:** Conceptualization (equal); Data curation (supporting); Formal analysis (equal); Investigation (equal); Methodology (equal); Supervision (lead); Validation (equal); Visualization (supporting); Writing‐original draft (supporting); Writing‐review & editing (equal).

## DATA AVAILABILITY STATEMENT

The dataset is available at the Dryad Digital Repository with DOI accession number: https://doi.org/10.5061/dryad.xgxd254gt

## Supporting information

Fig S1‐S2‐Table S1‐S4
