# Supplementary material for: High functional diversity in deep‐sea fish communities and increasing intraspecific trait variation with increasing latitude
Source: Ecol Evol. 2021 Jul 8;11(15):10600–12. doi: 10.1002/ece3.7871 (PMC8328419; doi:10.1002/ece3.7871)
Supplement: Supplementary file 1 — Fig S1‐S2‐Table S1‐S4 [file ECE3-11-10600-s001.docx]

Supplement 1: A description of raw morphological measurements taken from individual fishes identified in video footage (stereo-BRUVs) or from museum specimens (Fig. S1).


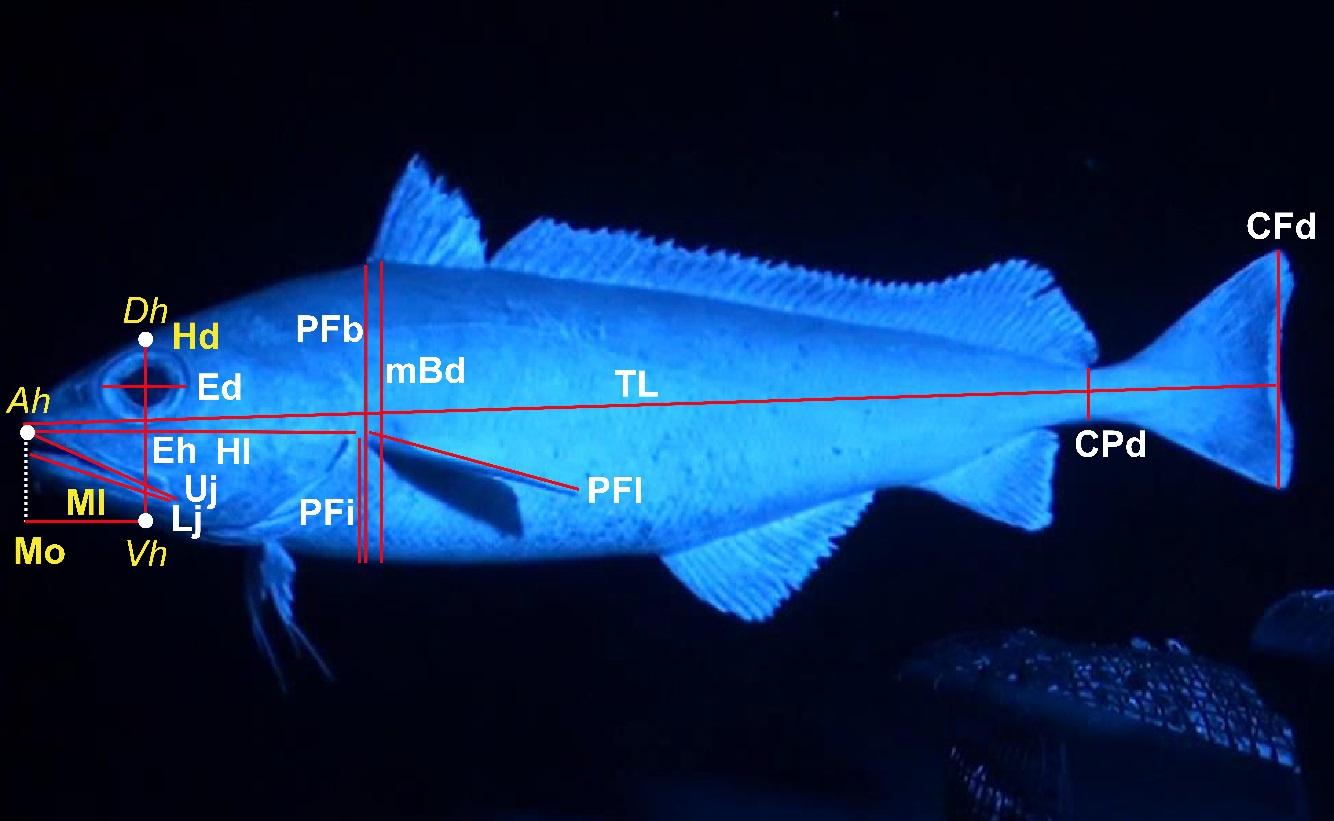


Figure S1. Raw morphological measurements obtained directly from stereo-BRUV footage; *CFd* = caudal fin depth, *CPd* = caudal peduncle depth, *Ed* = maximum eye diameter, *Eh* = eye height (length between the centre of the eye, and the white point denoted *Vh* (ventral head), *Hd* = head depth, *Hl* = head length, *Lj* = lower jaw length, *mBd* = maximum body depth, *Ml* = mouth length, *Mo* = mouth open (distance from the top of the mouth to the bottom of the head along the head depth axis), *PFb* = body depth at pectoral fin base, *PFi* = pectoral fin insertion, *PFl* = pectoral fin length, *TL* = total body length, *Uj* = upper jaw length. Note: *Mo, Hd,* and *Ml* (in yellow) are derived from a sequence of three measured 3D points within a single frame (*Dh* = dorsal head, *Ah* = anterior head, *Vh* = ventral head), and are obtained internally within EventMeasure software ([www.seagis.com.au](http://www.seagis.com.au/)), using the “Head Morphometrics” feature of EventMeasure version 4.4.

Supplement 2: Statistical analyses

*Univariate models*

Our primary aim was to model patterns of change in functional diversity metrics along the depth (or latitude) gradient and examine variations in these patterns with latitude (or depth). We therefore used linear mixed-effects models treating depth (or latitude) as a fixed factor and latitude (or depth) as a random factor. First, the best random-effects structure was chosen under a REML optimization (Zuur *et al.* 2009), using a small-sample corrected Akaike information criterion (AICc), from the following potential models: 1) a correlated random intercept and slope model, 2) an uncorrelated random intercept and slope model, and 3) a random intercept model. For this first step, we set the fixed model structure to include (normalised) linear, quadratic and cubic terms. Next, we set the random structure in place and performed the model selection considering the linear, quadratic and cubic terms as potential predictors, once again choosing a best model using AICc and under ML optimisation. Finally, we estimated a marginal R^2^ (variance explained by the fixed effect only) and a conditional R^2^ (attributable to fixed and random effects) (see Nakagawa & Schielzeth 2013; Johnson 2014; Nakagawa, Johnson & Schielzeth 2017). In addition, semipartial marginal R^2^ values were estimated for the different terms of the fixed effect (Jaeger *et al.* 2017). We used the nlme (Pinheiro *et al.* 2012), MuMIn (Barton 2009) and r2glmm (Jaeger 2017) R packages to perform the mixed modeling, the model selection and to estimate R^2^ values (marginal, conditional and semipartial).

Table S1. Parameter estimates, proportion of explained variance, and statistical significance of individual terms for the best models (using AICc) for each of 7 functional diversity metrics *vs* depth, using a linear mixed-effects model with latitude as a random effect. The best random structure for latitude (considering a random intercept model (RI), an uncorrelated random intercept and slope model (URIS), or correlated random intercept and slope model (CRIS)) and the best fixed effect (considering linear, quadratic and cubic terms for depth) were selected using AICc. VPFD and VNND were transformed to $y'={log}_{10}\left( y \right)$. The estimated standard deviation for the random slope in the CRIS model for FHV was 125.25 and the estimated correlation between the random intercept and slope was *r* = -0.75.

|  | **Metric** | **Best Random Structure** | **sd (**$\beta_{0}$**)** | **Marginal R^2^**  **(Fixed)** | **Conditional R^2^ (Fixed + Random)** | **Parameter** | **Semi-**  **partial**  **R^2^** | **Value** | **Std.Error** | **df** | ***F*** | ***P*** |
| --- | --- | --- | --- | --- | --- | --- | --- | --- | --- | --- | --- | --- |
| *Richness* | **FHV** | CRIS | 139.28 | 0.03 | 0.56 | Intercept | - | 400.0 | 58.30 | 39 | 6.86 | **<0.01** |
|  |  |  |  |  |  | Depth |  | 49.66 | 53.87 | 39 | 0.92 | 0.36 |
| *Dispersion* | **MPFD** | RI | 0.28 | 0.07 | 0.36 | Intercept | - | 3.57 | 0.12 | 39 | 29.30 | **<0.01** |
|  |  |  |  |  |  | Depth | - | 0.14 | 0.06 | 39 | 2.34 | **0.02** |
|  | **MNND** | RI | 0.1 | 0.33 | 0.41 | Intercept | - | 2.35 | 0.07 | 38 | 31.40 | **<0.01** |
|  |  |  |  |  |  | Depth | 0.331 | 0.23 | 0.04 | 38 | 5.09 | **<0.01** |
|  |  |  |  |  |  | Depth^2^ | 0.086 | -0.1 | 0.04 | 38 | -2.21 | **0.03** |
| *Regularity* | **VPFD** | RI | 0.16 | <0.001 | 0.32 | Intercept | - | 0.04 | 0.7 | 39 | 0.6 | 0.54 |
|  |  |  |  |  |  | Depth | - | -0.02 | 0.03 | 39 | -0.73 | 0.47 |
|  | **VNND** | RI | 0.18 | 0.05 | 0.35 | Intercept | - | -0.28 | 0.08 | 38 | -3.18 | **<0.01** |
|  |  |  |  |  |  | Depth | 0.013 | 0.03 | 0.04 | 38 | 0.93 | 0.36 |
|  |  |  |  |  |  | Depth^2^ | 0.047 | -0.07 | 0.04 | 38 | -1.81 | 0.07 |
| *Intra-specific Trait Variability* | **MPFD.I** | URIS | 0.12 | 0.32 | 0.45 | Intercept | - | 1.95 | 0.03 | 35 | 50.81 | **<0.01** |
|  |  |  |  |  |  | Depth | - | 0.19 | 0.06 | 35 | 3.12 | **<0.01** |
|  | **Prop.I** | URIS | 0.08 | 0.01 | 0.40 | Intercept | - | 0.34 | 0.01 | 34 | 21.52 | **<0.01** |
|  |  |  |  |  |  | Depth | - | -0.01 | 0.03 | 34 | -0.40 | 0.68 |

Table S2. Parameter estimates, proportion of explained variance, and statistical significance of individual terms for the best models (using AICc) for each of 7 functional diversity metrics *vs* latitude, using a linear mixed-effects model with depth as a random effect. The best random structure for depth (considering a random intercept model (RI), an uncorrelated random intercept and slope model (URIS), or correlated random intercept and slope model (CRIS)) and the best fixed effect (considering linear, quadratic and cubic terms for depth) were selected using AICc. VPFD and VNND were transformed to $y'={log}_{10}\left( y \right)$.

|  | **Metric** | **Best Random Structure** | **sd (**$\beta_{0}$**)** | **Marginal R^2^**  **(Fixed)** | **Conditional R^2^ (Fixed + Random)** | **Parameter** | **Semi-**  **partial**  **R^2^** | **Value** | **Std.Error** | **df** | ***F*** | ***P*** |
| --- | --- | --- | --- | --- | --- | --- | --- | --- | --- | --- | --- | --- |
| *Richness* | **FHV** | URIS | 55.38 | 0.19 | 0.23 | Intercept | - | 400.34 | 31.93 | 39 | 12.54 | **<0.01** |
|  |  |  |  |  |  | Latitude | - | -109.58 | 38.55 | 39 | -2.84 | **<0.01** |
| *Dispersion* | **MPFD** | RI | 0.10 | 0.23 | 0.27 | Intercept | - | 3.58 | 0.07 | 39 | 47.95 | **<0.01** |
|  |  |  |  |  |  | Latitude | - | -0.25 | 0.06 | 39 | -3.81 | **<0.01** |
|  | **MNND** | RI | 0.21 | 0.10 | 0.42 | Intercept | - | 2.19 | 0.10 | 37 | 21.44 | **<0.01** |
|  |  |  |  |  |  | Latitude | 0.017 | 0.11 | 0.10 | 37 | 1.1 | 0.28 |
|  |  |  |  |  |  | Latitude^2^ | 0.055 | 0.09 | 0.04 | 37 | 2.02 | **0.05** |
|  |  |  |  |  |  | Latitude^3^ | 0.055 | -0.10 | 0.05 | 37 | -2.04 | **0.05** |
| *Regularity* | **VPFD** | RI | <0.01 | 0.25 | 0.25 | Intercept | - | 0.04 | 0.03 | 39 | 1.28 | 0.2 |
|  |  |  |  |  |  | Latitude | - | -0.14 | 0.03 | 39 | -3.94 | **<0.01** |
|  | **VNND** | RI | 0.07 | 0.26 | 0.32 | Intercept | - | -0.35 | 0.04 | 39 | -7.38 | **<0.01** |
|  |  |  |  |  |  | Latitude | - | -0.16 | 0.03 | 39 | -4.26 | **<0.01** |
| *Intra-specific Trait Variability* | **MPFD.I** | RI | 0.2 | 0.01 | 0.35 | Intercept | - | 1.96 | 0.08 | 35 | 22.26 | **<0.01** |
|  |  |  |  |  |  | Latitude | - | 0.04 | 0.04 | 35 | 1.06 | 0.3 |
|  | **Prop.I** | URIS | 0.03 | 0.11 | 0.21 | Intercept | - | 0.34 | 0.01 | 34 | 20.75 | **<0.01** |
|  |  |  |  |  |  | Latitude | - | 0.04 | 0.02 | 34 | 1.83 | 0.07 |

Table S3: Parameter estimates, proportion of explained variance, and statistical significance of individual terms for the linear models testing the interaction between depth and latitude. Adj.R^2^ = Adjusted R squared, Prop.Expl.Var = Proportion of explained variance.

| **Metric** | **Parameter** | **Estimate** | **Std.Error** | **T.value** | **P.value** | **Adj.R^2^** | **Prop.Expl.Var** |
| --- | --- | --- | --- | --- | --- | --- | --- |
| **FVH** | Intercept | 404.413 | 30.892 | 13.091 | 0 | 0.273 | NA |
|  | Depth | 49.953 | 31.309 | 1.595 | 0.118 | 0.273 | 4.09 |
|  | Depth:Lat | 78.492 | 32.43 | 2.42 | 0.02 | 0.273 | 9.259 |
|  | Latitude | -104.274 | 31.249 | -3.337 | 0.002 | 0.273 | 18.684 |
| **MPFD** | Intercept | 3.593 | 0.061 | 59.153 | 0 | 0.335 | NA |
|  | Depth | 0.154 | 0.062 | 2.498 | 0.016 | 0.335 | 9.543 |
|  | Depth:Lat | 0.13 | 0.064 | 2.046 | 0.047 | 0.335 | 6.053 |
|  | Latitude | -0.236 | 0.061 | -3.835 | 0 | 0.335 | 22.236 |
| **VPFD** | Intercept | 0.051 | 0.035 | 1.467 | 0.15 | 0.301 | NA |
|  | Depth | -0.023 | 0.035 | -0.647 | 0.521 | 0.301 | 0.538 |
|  | Depth:Lat | 0.083 | 0.036 | 2.284 | 0.027 | 0.301 | 7.928 |
|  | Latitude | -0.142 | 0.035 | -4.052 | 0 | 0.301 | 26.165 |
| **MPFD.I** | Intercept | 1.959 | 0.043 | 46.099 | 0 | 0.322 | NA |
|  | Depth | 0.191 | 0.041 | 4.613 | 0 | 0.322 | 34.783 |
|  | Depth:Lat | -0.02 | 0.043 | -0.461 | 0.648 | 0.322 | 0.343 |
|  | Latitude | 0.05 | 0.046 | 1.068 | 0.292 | 0.322 | 1.889 |
| **Prop.I** | Intercept | 0.347 | 0.017 | 20.88 | 0 | 0.219 | NA |
|  | Depth | -0.006 | 0.016 | -0.37 | 0.713 | 0.219 | 0.13 |
|  | Depth:Lat | -0.045 | 0.017 | -2.692 | 0.011 | 0.219 | 13.811 |
|  | Latitude | 0.047 | 0.018 | 2.616 | 0.013 | 0.219 | 13.63 |

Table S4: Estimate and significance of the smoothing terms and the tensor product testing the interaction between depth and latitude using generalized additive models for MNND and VNND. In the “Parameters” column “te(Depth,Latitude)” represents the tensor product smooth term between depth and latitude. The smooth terms were constrained to a maximum of 3 degrees of freedom. edf = effective degrees of freedom, r.sq = R squared, Dev.expl = Explained deviance.

| **Metric** | **Parameters** | **edf** | **F** | **p.value** | **r.sq** | **Dev.expl** |
| --- | --- | --- | --- | --- | --- | --- |
| MNND | s(Depth) | 1 | 9.923 | 0.003 | 0.404 | 0.471 |
| MNND | s(Latitude) | 1.67 | 0.983 | 0.317 | 0.404 | 0.471 |
| MNND | te(Depth,Latitude) | 2.498 | 2.905 | 0.092 | 0.404 | 0.471 |
| VNND | s(Depth) | 1 | 0.808 | 0.374 | 0.355 | 0.425 |
| VNND | s(Latitude) | 1 | 0.058 | 0.81 | 0.355 | 0.425 |
| VNND | te(Depth,Latitude) | 2.94 | 1.946 | 0.12 | 0.355 | 0.425 |


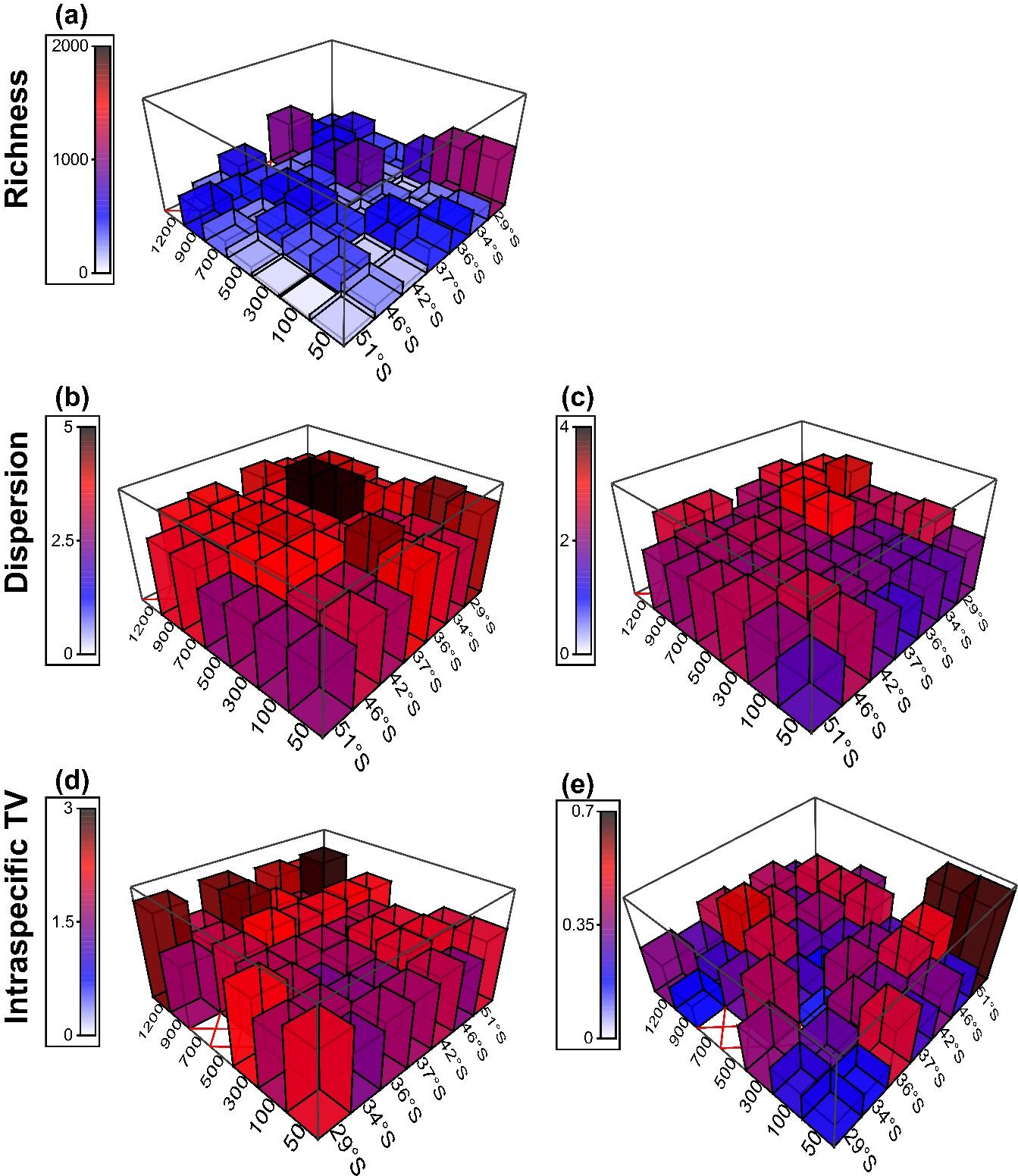


Figure S2. Three-dimensional shade plots showing the potential interactions between depth and latitude for interspecific metrics (a) FHV = functional hypervolume, (b) MPFD = mean pairwise functional distance, (c) MNND = mean nearest neighbour distance, and metrics documenting intraspecific trait variability (d) MPFD.I = intraspecific mean pairwise functional distance, and (e) Prop.I = proportion of variance explained by individual (intraspecific) variation.


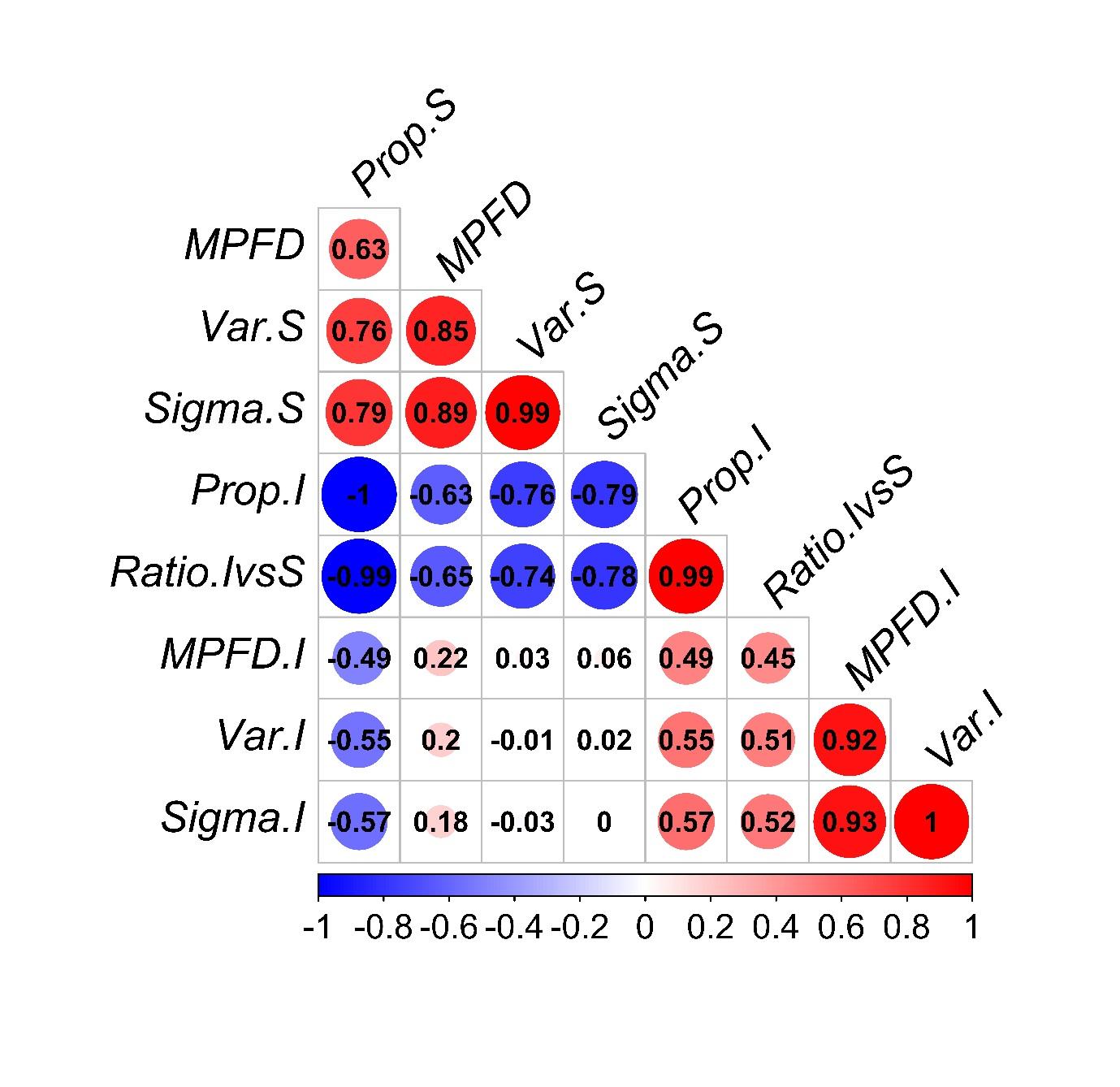
Figure S3. Heat map showing Pearson correlations among measures related to the partitioning of variance using a PERMANOVA approach whereby Var.S = variation in traits among different species, Sigma.S = square root of Var.S, Prop.I = proportion of total trait variation attributable to variation among individuals (var.I / (var.S + var.I)), Ratio.IvS = Ratio of individual versus species-level variation (Sigma.I / Sigma.S), MPFD.I = mean pairwise distance between all pairs of individuals belonging to the same species, Var.I = variation in traits among the individuals within a species, Sigma.I = square root of Var.I, and the functional metric, MPFD = mean pairwise functional distance.


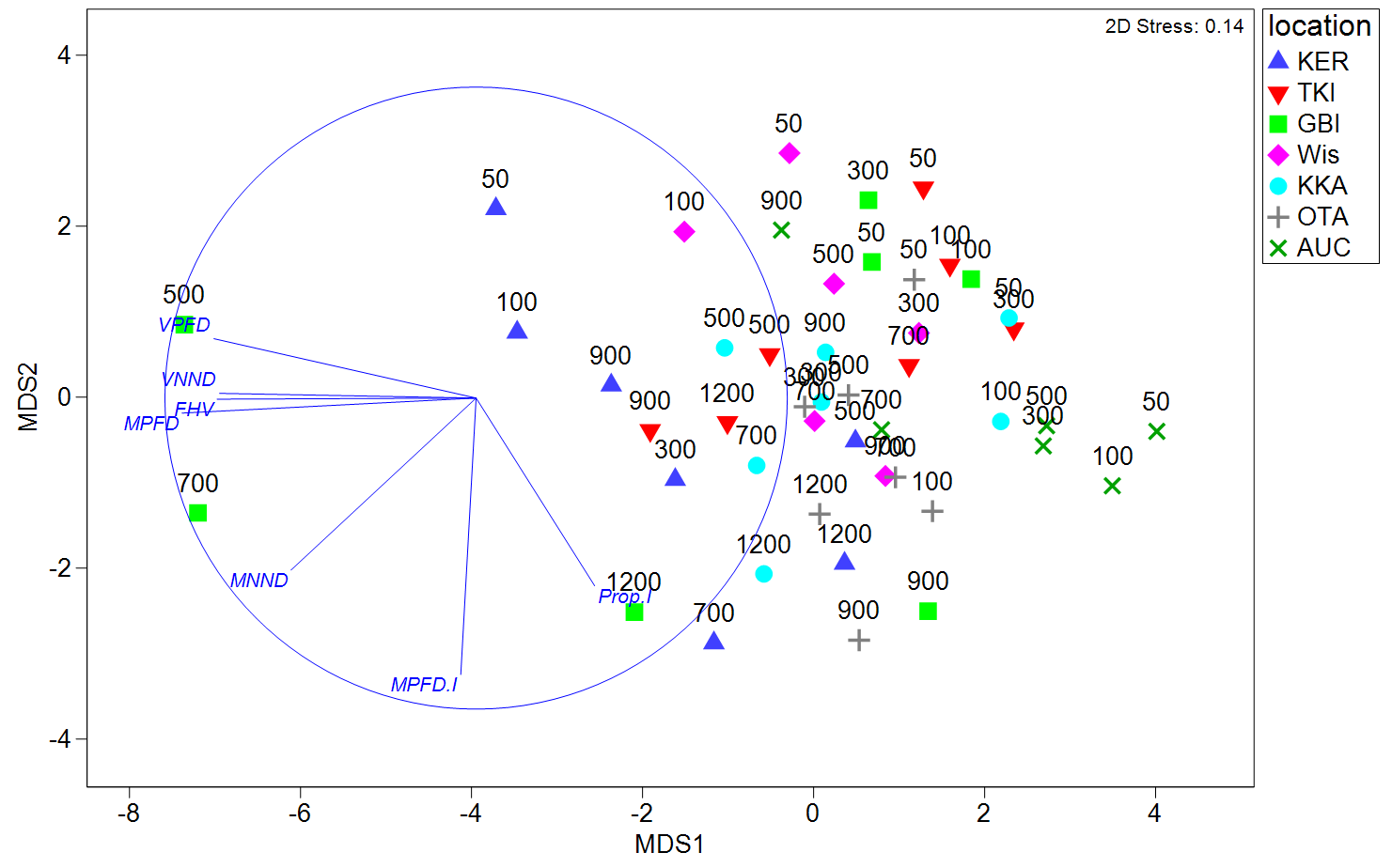
Figure S4. Metric multi-dimensional scaling (mMDS) ordination on the basis of Euclidean distances among depth-by-location centroids for *p* = 7 functional diversity metrics: functional hypervolume (FHV), mean pairwise functional distance (MPFD), variance of mean pairwise functional distance (VPFD), mean nearest neighbour distance (MNND), variance of mean nearest neighbour distance (VNND), mean pairwise distance, considering only intra-specific distances (MPFD.I), proportion of total trait variation attributable to variation among individuals (Prop.I). Labels represent depth (50 m – 1200 m) and symbols represent location (ordered here from north to south): KER = Kermadec Islands, TKI = Three Kings Islands, GBI = Great Barrier Island, WIs = White Island, KKA = Kaikōura, OTA = Otago, and AUC = Auckland Islands.


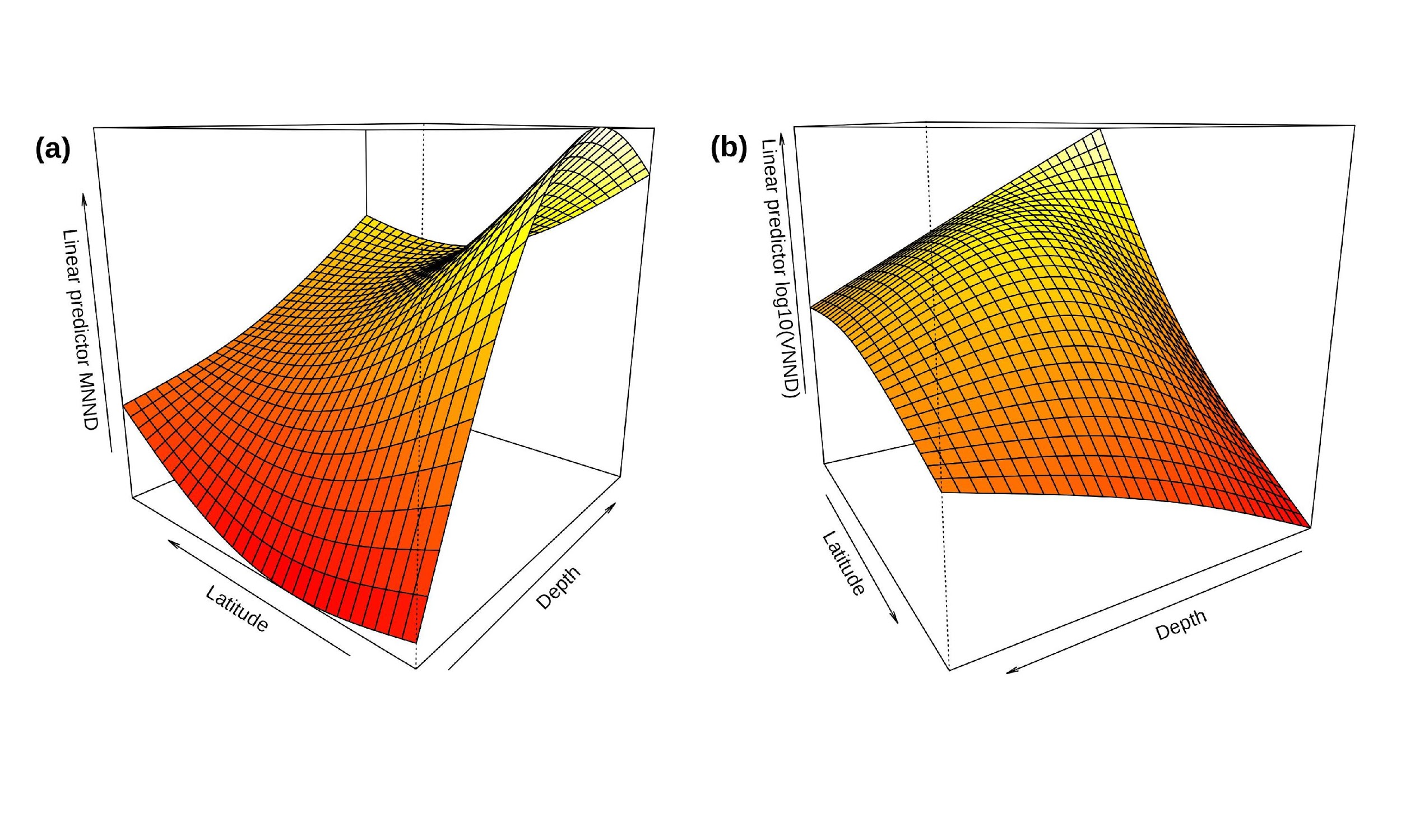
Figure S5: Three dimensional plots showing the shape of the interaction between depth and latitude for (a) MNND and (b) VNND (log10 transformed) via a tensor product. The tensor products were fitted with generalized additive models with smooth terms constrained to a maximum of 3 degrees of freedom to avoid overfitting.

**References**

Barton, K. (2009) MuMIn: multi-model inference. R package version 1. 0. 0. *http://r-forge. r-project. org/projects/mumin/*.

Jaeger, B. (2017) R2glmm: computes R squared for mixed (multilevel) models. *R package version 0.1,* **2**.

Jaeger, B.C., Edwards, L.J., Das, K. & Sen, P.K. (2017) An R 2 statistic for fixed effects in the generalized linear mixed model. *Journal of Applied Statistics,* **44,** 1086-1105.

Johnson, P.C. (2014) Extension of Nakagawa & Schielzeth's R2GLMM to random slopes models. *Methods in Ecology and Evolution,* **5,** 944-946.

Nakagawa, S., Johnson, P.C. & Schielzeth, H. (2017) The coefficient of determination R 2 and intra-class correlation coefficient from generalized linear mixed-effects models revisited and expanded. *Journal of the Royal Society Interface,* **14,** 20170213.

Nakagawa, S. & Schielzeth, H. (2013) A general and simple method for obtaining R2 from generalized linear mixed‐effects models. *Methods in Ecology and Evolution,* **4,** 133-142.

Pinheiro, J., Bates, D., DebRoy, S., Sarkar, D. & Team, R.C. (2012) nlme: Linear and nonlinear mixed effects models. *R package version,* **3**.

Zuur, A., Ieno, E.N., Walker, N., Saveliev, A.A. & Smith, G.M. (2009) *Mixed effects models and extensions in ecology with R*. Springer Science & Business Media.
